# How resistance-nodulation-division (RND) efflux pumps contribute to virulence

**DOI:** 10.1371/journal.ppat.1014228

**Published:** 2026-05-14

**Authors:** Corrella S. Detweiler, Ilyas Alav

**Affiliations:** 1 Department of Molecular, Cellular, and Developmental Biology, University of Colorado, Boulder, Colorado, United States of America; 2 Sir William Dunn School of Pathology, University of Oxford, Oxford, United Kingdom; Dartmouth Medical School: Dartmouth College Geisel School of Medicine, UNITED STATES OF AMERICA

## Abstract

In Gram-negative bacteria, multi-subunit efflux pumps transport molecules across the inner membrane, periplasm, and outer membrane to the extracellular environment. These efflux pumps include the Resistance-Nodulation-Cell Division (RND) superfamily, which utilize the membrane proton motive force to export a wide range of substrates against a concentration gradient. RND efflux pumps have been extensively studied for their fundamental role in the export of antibiotics, but they also play multifaceted roles in bacterial physiology. Notably, they are required for pathogen survival in the mammalian host when antibiotics are absent, an emerging aspect of their biology that is not well understood. Here, we analyze the evidence supporting several intertwined mechanistic hypotheses regarding the requirement for RND efflux pumps during infection. To do this, we explore why host- and bacterial-derived substrates need to be exported during pathogenesis, and the effects of proton translocation from the periplasm to the cytosol. We close by highlighting knowledge gaps and directions for future work regarding the role of RND efflux pumps in bacterial virulence.

## Introduction

Resistance-Nodulation-Division (RND) efflux pumps are an evolutionarily ancient superfamily of membrane transporter proteins and are necessary for virulence in all Gram-negative bacterial pathogens in which they have been studied [1]. Structurally, they are composed of a trimeric inner membrane RND transporter, a hexameric periplasmic adaptor protein, and a trimeric outer membrane channel protein [2]. RND efflux pumps actively transport substrates against concentration gradients by using energy from the proton motive force, which is generated as protons flow down their electrochemical gradient from the periplasm into the cytosol, across the inner membrane [3]. The coupling of proton movement to substrate export allows RND efflux pumps to expel substrates efficiently to the extracellular environment [4]. This also means that RND efflux pumps modulate the movement of protons across the inner membrane, potentially affecting the pH of the cytosol.

The RND superfamily consists of several families, with the Hydrophobe/Amphiphile Efflux 1 (HAE-1) family possessing the broadest substrate range, including antimicrobials, detergents, dyes, and solvents [5,6]. The polyspecificity of HAE-1 RND efflux pumps also contributes to their role in multidrug resistance of clinically important Gram-negative pathogens [7,8]. The expression and activity of RND efflux pumps is tightly regulated at a local and global level. For example, the AcrAB-TolC efflux pump is regulated by its local repressor AcrR, global transcriptional activators RamA, MarA, SoxS, and the two-component system EvgAS [9–13]. RND efflux pumps moreover export molecules that would be otherwise sensed by diverse regulators in the bacterial cell [14]. Therefore, the expression of RND efflux pumps is highly interconnected with the global regulatory network of bacterial cells.

**Fig 1:**
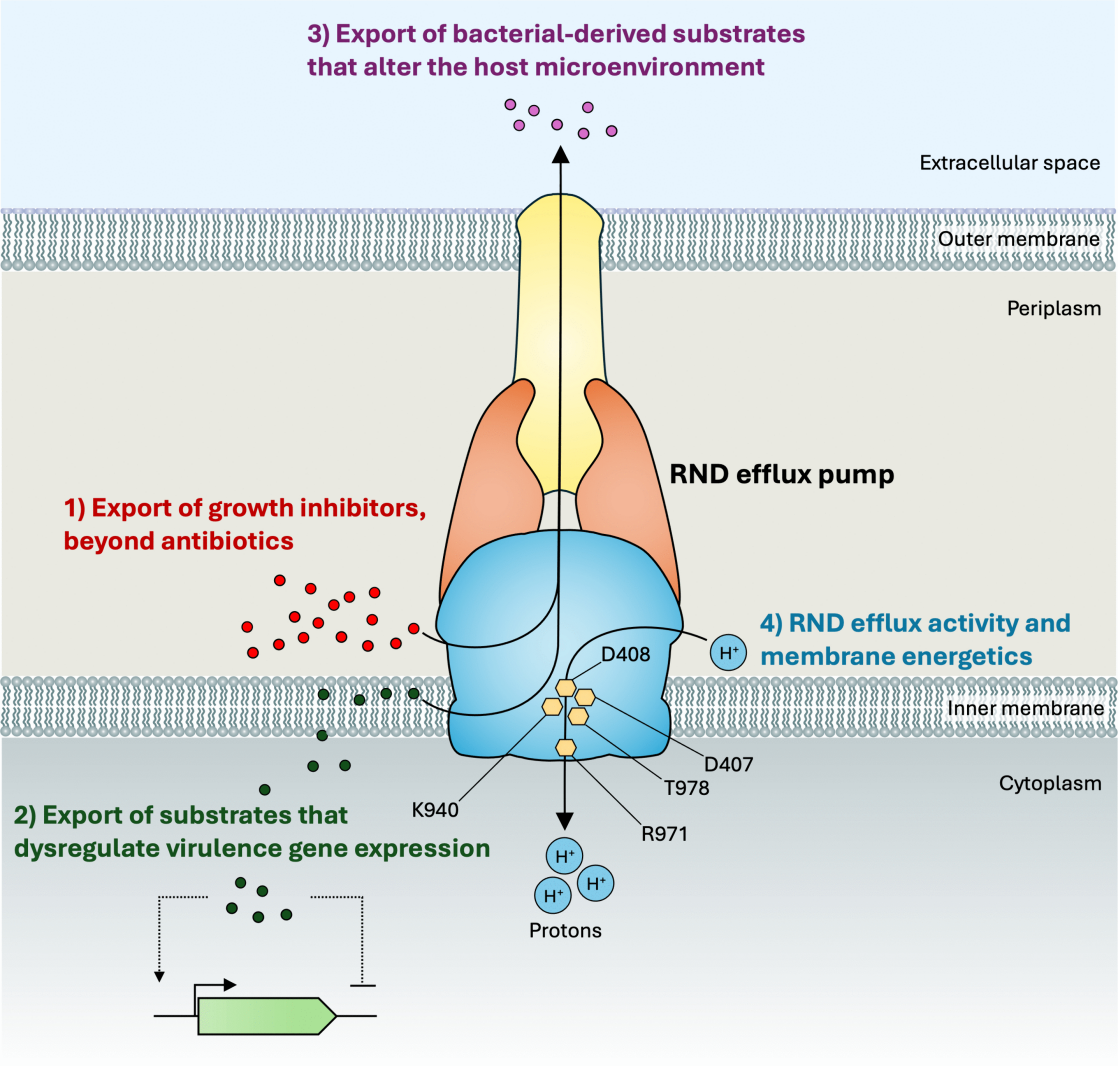
Schematic diagram highlighting the four hypotheses on how RND efflux pumps potentially contribute to virulence. **Hypothesis 1:** Substrates that inhibit the growth or survival of bacteria during infection are exported by RND efflux pumps, typically, from the periplasm or the outer leaflet of the inner membrane. This class of substrates includes antimicrobial peptides, bile salts, and steroidal hormones. **Hypothesis 2:** Certain molecules need to be exported by RND efflux pumps to prevent dysregulation of virulence genes. Accumulation of these substrates during infection is detected within the periplasm or possibly within the cytosol by spatiotemporal regulators of transcription. **Hypothesis 3:** RND efflux pumps export substrates, such as quorum-sensing molecules and siderophores, that modulate the microenvironment to favor bacterial growth and survival during infection. **Hypothesis 4:** The export of substrates by RND efflux pumps is concurrent with the translocation of protons from the periplasm to the cytosol. This could affect the local pH and membrane potential in the vicinity of RND efflux pumps and therefore bacterial physiology during infection. The residues of the proton relay network are denoted by orange hexagons, D407, D408, K940, R971, and T978.

The role of HAE-1 RND efflux pumps in virulence has been demonstrated in multiple Gram-negative bacteria [1]. However, their contribution to bacterial physiology during infection is likely complicated and multifactorial, and possible substrates associated with virulence remain unclear. The purpose of this review is to evaluate the evidence supporting mechanisms by which RND efflux pumps could contribute to bacterial virulence (Fig 1). We propose four hypotheses structured around why substrates need to be exported by RND efflux pumps during infection. The first hypothesis examines host- or bacterial-derived RND efflux pumps substrates that will inhibit bacterial growth if they are not expelled from the cell. The second hypothesis concerns the need to export substrates that dysregulate virulence gene expression. The third hypothesis explores substrates that are exported to modify the host microenvironment and enable virulence. The fourth and last hypothesis speculates on the effects of the movement of protons from the periplasm to the cytosol that is a consequence of export via RND efflux pumps.

## Hypothesis 1—Export of growth inhibitors, beyond antibiotics

The majority of known RND efflux pump substrates have been identified by their ability to inhibit bacterial growth or survival in the absence of efflux pumps or of efflux pump activity. Most of these studies have been carried out in standard media supplemented with a growth-inhibitory molecule. We define standard media as Luria-Bertani (LB) broth, Mueller–Hinton (MH) broth, or any other type of media used for the routine cultivation of bacteria. Accordingly, many RND efflux pump substrates studied to date are clinically relevant antibiotics. However, there is accumulating evidence that RND efflux pumps also export growth-inhibitory substrates that are host- or bacterial-derived, as revealed by studies of RND efflux pumps under conditions that are more infection-relevant.

### Export of host-derived bacterial growth inhibitors

As the first line of defense against invading pathogens, the innate immune system is highly conserved amongst vertebrate and invertebrate animals [15]. The cells that comprise the innate immune system produce a range of soluble effector molecules that kill or prevent the growth of invading pathogens. These antimicrobial molecules are not pathogen-specific and target a range of microbes [16]. Bacteria possess several mechanisms, including export by RND efflux pumps, to resist host-produced antimicrobial factors [17]. In the following section, we evaluate studies that suggest a role for RND efflux pumps in protecting bacterial cells from host antimicrobial factors.

#### Antimicrobial peptides.

Antimicrobial peptides (AMPs) are an evolutionarily conserved group of key host effector molecules with broad-spectrum antimicrobial activity. In vertebrates, AMPs are produced by circulating immune cells such as macrophages and neutrophils [18], and site-specific epithelial cells, such as Paneth cells in the small intestine and keratinocytes in the epidermis [19,20]. Invertebrate AMPs are produced in various tissues and cells, including the gut and respiratory organs, hemolymph, and immune cells [21,22]. Structurally, AMPs are short peptides, between 10 and 50 residues, that generally have an overall cationic charge [23]. AMPs have multiple mechanisms of action, but cationic AMPs typically interact with the negatively charged bacterial membrane, resulting in increased membrane permeability, cell membrane lysis, and ultimately causing bacterial cell death [24,25]. While colistin and polymyxin B are model AMPs, they are not host-produced and so will not be discussed in this section.

The causative agent of gonorrhea, *N. gonorrhoeae,* is the most extensively studied pathogen regarding the role of RND efflux pumps in AMP resistance. Overall, the data indicate that the sole *Neisseria* RND efflux pump exports AMPs, but that not all AMPs are exported by MtrCDE. Shafer and colleagues [26] showed that the inactivation of *mtrD*, encoding the RND-transporter component of MtrCDE, reduced bacterial growth in gonococcal base liquid medium supplemented with structurally diverse AMPs, including the porcine cathelicidin peptide PG-1, human cathelicidin peptide LL-37, and the horseshoe crab-derived peptide TP-1. The loss of *mtrD* had no impact on growth-inhibition by human defensin HNP-2, indicating MtrCDE does not capture all classes of AMP [26]. In a different study, deletion of *mtrE* significantly reduced *N. gonorrhoeae* survival when exposed to LL-37 or bactericidal/permeability-increasing (BPI) protein in gonococcal base liquid medium, a phenotype rescued by complementation [27]. In the closely related pathogen *Neisseria meningitidis*, a causative agent of meningitis, inactivation of *mtrD* also reduced growth in broth supplemented with LL-37 or PG-1 [28]. These data suggest that the MtrCDE pump plays an important role in *Neisseria* in the export of LL-37, and possibly BPI. Lyu and colleagues (161) solved the crystal structure of the *N. gonorrhoeae* MtrD RND-transporter in complex with LL-37 (residues 17–32), indicating that LL-37 is a substrate for the MtrCDE efflux pump. In conclusion, *Neisseria* appears to require the sole RND efflux pump it encodes to export some, but not all, AMPs.

In contrast to the single RND efflux pump in *Neisseira*, Enterobacterales encodes multiple RND efflux pumps, of which the AcrAB-TolC role in AMP efflux has been studied in *K. pneumoniae* and *E. coli.* Padilla and colleagues [29] found that deletion of *acrB* in *K. pneumoniae* decreased survival in the presence of human defensins HNP-1, HBD-1, and HBD-2. As capsular polysaccharide and lipopolysaccharide can affect AMP susceptibility [30], they also measured expression of both polysaccharides and found no difference between the *acrB*-deletion mutant and wild-type strain, suggesting a direct role for AcrAB-TolC in AMP export. In *E. coli*, differences in growth media significantly impact whether RND efflux pumps can export AMPs. Rieg and colleagues [31] reported that in MHB, deletion of *acrAB* in *E. coli* did not affect survival upon exposure to LL-37, HNP-1,2 and 3, HD-5, or hBD-2. In contrast, Warner and Levy [32] showed that the loss of *acrAB* significantly reduced survival in LB supplemented with LL-37 and HBD-1. These differences may reflect that LB is typically higher in salt, which influences AMP activity, than MHB [33,34]. These data highlight that a role for AcrAB-TolC in AMP export can be influenced by experimental setup and conditions, therefore, care must be taken when interpreting results. Nevertheless, the data overall indicate that RND efflux pumps can export AMPs.

#### Bile acids and salts.

Bile acids and their conjugated versions (bile salts) are synthesized in the liver from cholesterol, which are then secreted into bile and stored in the gallbladder [35]. They primarily function as surfactants to aid in the digestion of dietary fats but also serve as antimicrobials against invading pathogens in the gut environment by inhibiting bacterial growth [35–37]. Enteric pathogens have evolved to recognize bile acids and salts as signals for the regulation of virulence factors and adaptive stress responses, to survive within the bile-rich gastrointestinal tract [38–40]. In addition, enteric pathogens require RND efflux pumps to export bile acids and salts that compromise growth, discussed below, and because they interfere with virulence gene expression, discussed under Hypothesis 2.

In Enterobacterales, the AcrAB-TolC efflux pump plays a crucial role in bile acid and salt resistance, with AcrAB contributing to the active efflux of bile salts [41,42]. Further studies confirmed that inactivation or deletion of *acrAB* in *E. coli* significantly reduced growth in broth or on agar containing sodium deoxycholate [43,44]. The structure of the *E. coli* AcrB transporter in complex with sodium deoxycholate provided further evidence that bile salts are indeed substrates of the AcrAB-TolC pump [44]. In the closely related pathogen *S. flexneri*, exposure to bile salts significantly increased *acrB* expression and deletion of *acrB* impaired *S. flexneri* growth in 0.4% or 2% bile salts, which was rescued upon complementation [45]. In the lung pathogen *K. pneumoniae*, inactivation of *acrA* or *acrB* also significantly impaired growth and reduced survival in broth containing crude bile or various bile salts, compared to wild type [46–48]. Whether bile acid resistance facilitated *K. pneumoniae* colonization of the lung, into which bile acids can be aspirated, is unclear. Nevertheless, these studies emphasize the crucial role of AcrAB in protecting Enterobacteriales from bile acids and salts.

In *S.* Typhimurium, there is intriguing evidence that in the absence of AcrAB, other RND efflux pumps can also export bile salts if they are overexpressed. In a strain lacking *acrAB,* susceptibility to sodium deoxycholate was not impacted by the deletion of other RND efflux genes. However, plasmid-mediated overexpression of *acrD*, *acrEF*, or *mdtABC* RND efflux genes in the Δ*acrAB* mutant restored growth at higher concentrations of sodium deoxycholate, like the wild-type strain [49]. In addition to bile resistance, AcrAB is required by *S.* Typhimurium for bile adaptation. Urdaneta and Casadeús [50] found that mutant strains lacking *acrA*, *acrB*, or *tolC* exposed to sublethal concentrations of sodium deoxycholate failed to grow on agar supplemented with higher concentrations of sodium deoxycholate or ox bile after 1, 2, and 3 days [50]. In contrast, mutant strains with deletions in other RND efflux genes adapted to sodium deoxycholate or ox bile as well as the wild-type strain, suggesting a critical role for AcrAB in bile adaptation [50]. In other words, *S.* Typhimurium RND efflux pumps have overlapping functions but are not interchangeable.

Other prominent enteric pathogens outside of Enterobacterales order also require RND efflux pumps to grow in the presence of bile acids and salts. In *C. jejuni*, the CmeABC efflux pump is essential for resistance to and growth in the presence of bile acids and salts. Lin and colleagues [51] demonstrated that mutant *C. jejuni* strains lacking *cmeB* and/or *cmeC* had 32–128-fold lower MICs to various bile salts compared to wild-type, which were rescued upon complementation. Furthermore, the deletion of *cmeB* and/or *cmeC* completely inhibited the growth of *C. jejuni* in broth supplemented with sodium choleate just after 3 hours [52]. These data indicate that CmeABC is necessary for the survival of *C. jejuni* during colonization of bile-rich environments such as the small intestine. In *H. pylori*, the inactivation of *hefC*, encoding an AcrB ortholog, significantly reduced survival in Ham’s F12 bovine serum albumin broth supplemented with sodium cholate, deoxycholate, or glycodeoxycholate after 16–18 hours [53]. *H. pylori* also infect the duodenum, which receives bile from the gallbladder and liver; therefore, HefC may protect *H. pylori* from bile in this infection niche. In *V. cholerae*, two different RND efflux pumps enable growth in the presence of bile salts. Bina and colleagues [54] found that deletion of both *vexB* and *vexD* (AcrB orthologs) was necessary to inhibit growth in LB broth supplemented with bile salts, suggesting RND efflux pump redundancy in this context. Thus, it appears that pathogens that colonize the upper gastrointestinal tract require RND efflux pumps to grow and survive in the presence of bile acids and salts.

#### Gonadal steroid hormones.

In vertebrates, gonadal steroid hormones regulate sexual behavior, traits, and development, and influence skeletal, cardiac, and immune systems [55]. There are three major classes of gonadal steroid hormones: androgens (includes testosterone), estrogens, and progestogens [55]. In addition to their significant physiological roles, some gonadal steroid hormones also exhibit antimicrobial activities, thereby functioning to protect host cells in the reproductive tract from invading pathogens [56,57]. There is evidence that RND efflux pumps in certain bacterial pathogens export gonadal steroid hormones, thereby protecting the bacteria from the antibacterial effects of the hormones.

As a prominent STI pathogen, *N. gonorrhoeae* is exposed to significant levels of gonadal steroid hormones during colonization and invasion of the genitourinary tract. An early study by Morse and colleagues [57] showed that progesterone concentrations 1000-fold higher than physiological levels inhibited the growth of *N. gonorrhoeae* and *N. meningitidis* by inhibiting enzymes involved in cellular respiration. Later, Jerse and colleagues [58] found that in *N. gonorrhoeae*, inactivation of *mtrD* (encoding inner membrane RND transporter MtrD) or *mtrE* (encoding outer membrane channel MtrE) significantly reduced growth on agar supplemented with progesterone compared to wild-type strain. There are no crystal structures of MtrD in complex with progesterone, but molecular dynamic simulations support spontaneous binding and entry of progesterone into the MtrD substrate pocket [59]. These data strongly suggest that the MtrCDE efflux pump exports progesterone. We note here that direct evidence that during human infections, RND efflux pumps contribute to bacterial virulence remains limited. For example, a controlled human male urethral challenge study with *N. gonorrhoeae* found that the MtrCDE efflux pump was not required for infection by strain FA1090 and did not provide a measurable competitive advantage in that setting [60]. However, these data contrast with earlier female mouse genital tract studies using strain FA19, in which MtrCDE-deficient *N. gonorrhoeae* had reduced fitness, and follow-up experiments suggested that this discrepancy was largely strain-dependent [58,60]. Moreover, the human challenge data also supported the existence of an early gonococcal colonization bottleneck [60]. Thus, while RND pumps can contribute to bacterial fitness and host adaptation in humans, their role in virulence during human infection needs additional interrogation with an emphasis on strain and infection site differences.

In *E. coli*, AcrAB-TolC has also been implicated in the efflux of gonadal steroid hormones. Elkins and Mullis [61] found that inactivation of *acrB* in *E. coli* reduced growth on LB agar supplemented with 256 µg/mL estradiol, progesterone, or hydrocortisone from 10 to 1,000-fold, compared to parent strain. Furthermore, the *acrB* mutant strain accumulated significantly more tritium-labeled estradiol, progesterone, and hydrocortisone, compared to wild-type. Complementation of the *acrB* mutant strain with either *acrB* or *mdtF* (encoding inner membrane RND transporter MdtF) on a high-copy number plasmid reduced the accumulation of the tritium-labeled steroid hormones. Interestingly, complementation with *mdtF* reduced steroid hormone accumulation more than *acrB*, suggesting that the MdtEF efflux pump also has the capacity to export gonadal steroid hormones. It again appears that RND efflux pumps can have overlapping functions that could be revealed or caused by overproduction of a specific efflux pump.

#### Other host-produced bacterial growth inhibitors.

The bacterial host produces a wide range of other molecules that affect bacterial physiology. Some of these have been found to inhibit the growth or survival of bacteria lacking RND efflux pump genes, suggesting a possible role for RND efflux pumps in their export. There is evidence that RND efflux pumps in several different species may export polyamines, organic compounds with two or more amino groups that play important physiological roles in bacteria. In *A. baumannii*, deletion of *adeB* caused an eight-fold and a two-fold reduction in spermine and spermidine MIC, respectively [62]. In *E. coli,* loss of *acrB* caused a two-fold reduction in spermidine MIC, suggesting slight increased susceptibility [63]. In *B. pseudomallei*, deletion of *bpeAB* caused a five-fold reduction in extracellular levels of *N*-acetylspermidine and accumulation of three-fold higher levels of radiolabeled spermidine compared to wild-type, which was reversed upon complementation [64]. While there are polyamine-specific transporter proteins, RND efflux pumps likely support the efflux of polyamines from the periplasm to the extracellular environment.

While many of these experiments utilize host-derived substrates at concentrations higher than are consistent with physiology, it is conceivable that multiple *in vivo* host substrates interact with RND efflux pumps simultaneously, increasing the effective growth inhibitory concentration of any given substrate. For instance, Morgan and colleagues has shown that the *A. baumannii* AdeB pump can bind multiple ethidium bromide molecules at a time [65]. AMPs can also simultaneously occupy multiple spaces within the substrate binding pocket, as a cryo-EM structure of *N. gonorrhoeae* MtrD bound a fully functional fragment of the host-derived LL-37 AMP (residues 17–32) at multiple sites [66].

### Bacterial-derived metabolites

Bacterial metabolites comprise structurally diverse substances that are intermediate or end products of metabolism. Although some metabolites diffuse out of bacterial cells, the majority are exported across one or both membranes by transport proteins as a means of maintaining intracellular homeostasis [67]. Research into bacterial metabolites has been gaining traction due increasing recognition of their importance and prevalence in host-microbiome interactions. While metabolomic studies have provided insight into the potential endogenous substrates of RND efflux pumps in Gram-negative bacteria, whether RND efflux pumps actively export metabolites, and the function of these metabolites overall, remain unclear.

#### Indoles produced by the microbiota can inhibit bacterial growth.

Indole is a compound found in the body of most mammals, produced by gut bacteria as a product of tryptophan metabolism [68]. While indole does not have a clear intracellular target, it appears to inhibit bacterial growth by altering membrane tension, increasing permeability of membranes to protons in a dose-dependent manner, and inhibiting cell division in *E. coli* [69]. Whether RND efflux pumps export indole has been contentious. An early study Kawamura-Sato and colleagues [70] reported that deletion of *acrEF*, encoding components of the AcrEF-TolC efflux pump, in *E. coli* increased intracellular accumulation of indole by two-fold compared to wild-type when grown in minimal synthetic media supplemented with 150 µM tryptophan. Additionally, the loss of *acrEF* reduced secretion of indole into the extracellular media, which was rescued by complementation. Interestingly, the loss of *acrAB* had no impact on indole accumulation or secretion. A later study found that in *E. coli*, the loss of *acrF* increased generation times when grown in LB broth supplemented with 3 mM indole compared to wild-type [71]. The loss of *acrE* reduced indole in the supernatant by ~40% compared to supernatant from wild-type strain after 8 hours of growth. Notably, the loss of *acrF* or *tolC* had no impact on indole levels in the supernatants compared to wild-type. After 24 hours of growth, there was no difference between the *acrE*, *acrF*, and *tolC* mutant strains compared to wild-type [71]. Therefore, the role of AcrEF-TolC in indole export remains to be established.

An indole derivative potentially exported by RND efflux pumps is melatonin, a hormone involved in the sleep-wake cycle of vertebrates. In *E. coli*, the inactivation of *acrB* has completely inhibited growth in M9 glucose media supplemented with 4 mg/mL melatonin [72]. While the melatonin concentration tested was significantly higher than peak melatonin levels in adult humans (100–200 pg/mL) [73], the data suggest that RND efflux pumps could have a role in exporting melatonin or other indole derivatives.

#### Secretion of bacterial factors as suggested by metabolomics.

Cauilan and colleagues [74] were the first to report a role for AcrAB-TolC in central metabolism using untargeted metabolomics. They compared the endometabolomes (intracellular metabolites) and exometabolomes (extracellular metabolites) of wild-type *E. coli* BW25113 and its Δ*acrB*, Δ*tolC*, and Δ*acrR* mutant derivatives grown in EZ rich defined medium. The supernatants, corresponding to the exometabolome, of the wild-type strain contained significantly higher levels of 2-ketoisovaleric, *N*-acetylaspartic, citric, succinic, and nonanoic acids compared to the Δ*acrB* strain [74], suggesting reduced export to the extracellular environment due to the loss of AcrB. The most notable difference between the Δ*acrR* and Δ*acrB* mutants was the shift in lysine levels: the Δ*acrR* strain, which over-expresses AcrAB-TolC, had lower intracellular and higher extracellular levels of lysine, whereas relative lysine concentrations were reversed in the Δ*acrB* strain. The authors suggested that AcrAB-TolC may be involved in the efflux of lysine or its intermediates, but this and other findings from the work have not been experimentally verified.

A complementary untargeted metabolomics profiling of wild-type *E. coli* MG1655 and *S.* Typhimurium SL1344 and their respective AcrB D408A loss-of-function mutants was carried out in MOPS minimal medium by Wang-Kan and colleagues [75]. As MOPS minimal media contains only salts, trace elements, and glucose as the sole carbon source, all metabolites must be synthesized *de novo*, removing the confounding factor of metabolite acquisition from the extracellular environment. Despite 95% protein sequence identity between *E. coli* and *S. Typhimurium* AcrB, the mutant strains in each species had distinct metabolic profile alterations relative to wild type. In the AcrB D408A *E. coli* mutant compared to wild type, the endometabolome contained more oxidized fatty acids, which are structurally similar to leukotrienes, lipoxins, prostaglandins, and thromboxanes. In the *S.* Typhimurium AcrB D408A endometabolome, metabolites related to sphingolipids and oxidized fatty acids were detected at significantly higher levels compared to wild type. These findings are intriguing in part because in *E. coli* and *S.* Typhimurium, AcrAB-TolC confers resistance to the long-chain fatty acid salts, sodium decanoate, and sodium dodecanoate [76,77]. While the potential AcrAB-TolC substrates identified by metabolomics were not validated, they warrant further exploration.

Metabolomics have also been recently applied to the study of RND efflux pumps in *P. aeruginosa* [78]. In a rich medium, oxidized fatty acids accumulated in the exometabolome of wild-type as compared with the Δ4*mex* mutant strain lacking *mexAB-oprM*, *mexCD-oprJ*, *mexEF-oprN*, and *mexXY*. Long-chain homoserine lactone quorum-sensing molecules were among the potential RND substrates identified [78]. Notably, complementation of the Δ4*mex* strain with individual RND efflux pumps produced specific exometabolomic profiles, suggesting that each RND efflux system exports a particular set of metabolites. These findings have not yet been validated, but the fact that oxidized fatty acids were identified in studies of *P. aeruginosa*, *E. coli*, and *S.* Typhimurium, suggests that this class of metabolites are likely RND efflux pump substrates requiring further investigation.

## Hypothesis 2—Export of substrates that dysregulate virulence gene expression

One reason RND efflux pumps are required for bacterial pathogenesis could be that, in their absence, virulence genes are inappropriately regulated during infection. Evidence suggests that periplasmic and cytosolic sensors can detect accumulated substrates of RND efflux pumps and, in response, dysregulate key virulence determinants. For example, during growth in MOPS minimal media, inactivation of the *acrB* gene in several different strains of *S.* Typhimurium significantly reduced the expression of genes within *Salmonella* Pathogenicity Island (SPI)-1 required for epithelial cell invasion. Complementation with *acrB* in *trans* restored SPI-1 gene expression to wild-type levels [79]. In the same nutrient-limited MOPS medium, Wang-Kan and colleagues [75,80] found that the non-functional AcrB D408A mutant *S.* Typhimurium SL1344 strain had significantly reduced expression of multiple virulence genes within SPI-1 and SPI-2 [80]. Interpretation of these data is challenging because during infection SPI-1 and SPI-2 are inversely regulated: SPI-1 is induced in and required for infection of the intestine, whereas SPI-2 is induced in and required for growth within macrophages, in which SPI-1 is repressed [81–83]. However, two-component regulatory systems (e.g., PhoPQ, PmrAB, EnvZ/OmpR, SsrAB) [81,84–86] activate and repress the SPIs, suggesting mechanisms by which RND efflux pumps could influence their expression. Substrates of RND efflux pumps, such as bile acids and salts, AMPs, and gonadal steroid hormones, can be sensed by periplasmic and/or cytosolic proteins [87–89]. For example, gonadal steroid hormones can bind to the cytosolic regulator MtrR, preventing it from being able to repress expression of *mtrCDE* [89]. It follows that in bacteria with compromised efflux pumps, key substrates accumulate to levels sufficient to change the transcription of virulence determinants, even at concentrations low enough to enable growth in broth. We outline below examples of sensor proteins in the cytosol and in the periplasm that respond to accumulated RND efflux pump substrates and reduce virulence gene expression. We focus on *S*. Typhimurium based on the abundance of data supporting the cytosolic and periplasmic detection of RND efflux pump substrates and their subsequent effects on virulence gene expression.

### The RamRA system detects RND efflux pump substrates that accumulate in the cytosol

**Fig 2:**
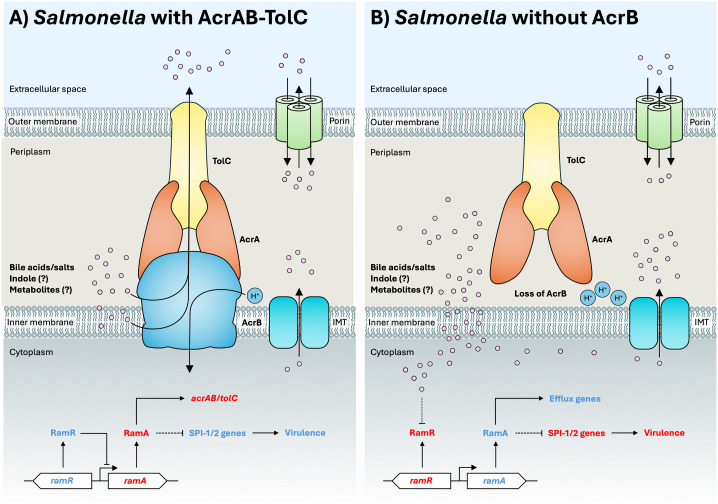
Potential mechanism of how the RamRA system regulates virulence in *Salmonella* Typhimurium. **A)** During infection with *S.* Typhimurium expressing an intact AcrAB-TolC efflux pump, exogenous host-derived (e.g., bile acids/salts) or microbiota-produced molecules (e.g., indole derivatives), denoted by pink circles could passively diffuse into the periplasm through porins. Simultaneously, possible endogenous AcrAB-TolC substrates derived from waste metabolites and metabolic intermediates are exported from the cytosol to the periplasm through inner membrane transporters (IMT) and/or passively diffuse across the inner membrane. Substrates captured by AcrAB-TolC are exported to the extracellular environment. Bile acids/salts, and possibly indole and metabolites, bind RamR to reduce its affinity for the *ramA* promoter, thereby relieving repression of *ramA.* Therefore, the levels of RamR inhibitor molecules are maintained at basal levels to regulate the repression of *ramA,* thus ensuring appropriate expression of *acrAB*-*tolC* and possibly *Salmonella* Pathogenicity Island (SPI)-1/2 genes. **B)** In *S.* Typhimurium without AcrB, during infection or in broth under host-like conditions, exogenous and endogenous substrates likely accumulate within the cytosol, periplasm, and the inner membrane. Substrates in the cytosol could interact with RamR, leading to the derepression of *ramA*. Increased RamA production stimulates the expression of efflux genes and likely inhibits genes within the SPI-1 and SPI-2, which would interfere with *S.* Typhimurium virulence in the gastrointestinal tract and in deep tissues, respectively, impairing virulence. Genes/proteins in red are downregulated and those in light blue are upregulated. Dashed lines indicate a possible but unproven link. IMT, inner membrane transporter.

RamA is a cytosolic AraC/XlyS family transcriptional activator of the *acrAB*-*tolC* genes in *K. pneumoniae* and *S*. Typhimurium [12,90]. RamA is a global regulator that has also been suggested to activate the expression of virulence genes within SPI-1 and SPI-2 of *S.* Typhimurium [91]. RamA is in turn regulated by its local repressor RamR, which itself is regulated by small molecule RND efflux pump substrates, including antimicrobial dyes, bile acids, and indole [88,92,93]. Binding of these small molecules to RamR decreases its affinity for the *ramA* promoter and derepresses RamA, which increases *acrAB*-*tolC* expression. The cytosolic efflux pump substrates that regulate RamR could reach the inner membrane or periplasm for capture by RND efflux pumps by diffusion, via inner membrane transporters, or by one of the RND efflux pumps, such as AcrAD-TolC, that ferry cytosolic substrates all the way across the cell envelope to the outside [94,95]. By effectively reducing the cytosolic concentration of substrates that bind RamR, RND efflux pumps indirectly maintain basal levels of *acrAB*-*tolC* expression and enable the appropriate regulation of virulence genes via RamA (Fig 2).

In the absence of AcrB, allosteric inhibitors of RamR may accumulate in the periplasm and the cytosol. The cytosolic inhibitors could prevent RamR from binding to the *ramA* promoter, resulting in its de-repression. In turn, the increased production of RamA would result in the activation of the *acrAB* promoter, as well as other genes within the RamA regulon. In *S.* Typhimurium, the loss of *acrB* or exposure to efflux inhibitors resulted in a significant upregulation of *ramA* [79,96], suggesting an important role for RamA in the upregulation of AcrAB-TolC in response to the loss of efflux function. Similarly, the loss of *acrAB* or inhibition of AcrAB in *E. coli* activated the *acrAB* promoter, mediated by the upregulation of *marA* and *soxS* (homologs of *ramA*) expression in the Δ*acrB* strain [14]. The activation of *acrAB* expression in *E. coli* was suggested to be due to the accumulation of metabolites from enterobactin, cysteine, and purine biosynthesis, and gluconeogenesis metabolic pathways, indicated by the reduction of Δ*acrB*-mediated induction of *acrAB* expression upon deletion of single genes in these pathways [14]. Therefore, the AcrAB-TolC efflux pump appears to regulate its own expression, possibly in response to the accumulation of its substrates which could affect the expression or activity of global transcriptional activators, such as MarA, RamA, and SoxS.

While the upregulation of global transcriptional activators like RamA confers an advantage to bacterial cells in response to stress, they may also dysregulate expression of virulence genes. In line with this thinking, *ramA* overexpression in *S.* Typhimurium causes downregulation of SPI-1 and 2 gene expression, reduces adhesion and intracellular survival within macrophages, and reduces colonization of *Caenorhabditis elegans* [91]. Interestingly, indole induces *ramA*, leading to induction of *acrB*, but repression of SPI-1 genes. This observation suggests that indole represses SPI-1 genes in a RamA-dependent manner [92] and could partly explain how deletion of *acrB* in *S.* Typhimurium reduces virulence gene expression, and possibly *in vitro* and *in vivo* colonization. Holden & Webber [97] hypothesized that increased expression of global activators such as *marA*, *ramA*, and *soxS* upon exposure to environmental stresses regulates RND efflux pump activity to allow detoxification of cells, however, this results in a trade-off in other phenotypes, such as biofilm formation and virulence [97]. Therefore, the downregulation of virulence genes in the absence of RND efflux pump gene expression could be a fitness trade-off for bacterial cells. Interestingly, in the non-functional AcrB D408A mutant strain of S. Typhimurium, the expression of virulence genes is significantly downregulated, but *ramA* is not upregulated [80]. While other RND efflux pumps also do not appear to be overexpressed in the AcrB D408A mutant, this does not seem to explain why *ramA* is not induced in the AcrB D408A mutant. Perhaps the presence of the AcrB D408A mRNA, protein, or efflux pump is detected directly or indirectly by RamA, or proton translocation into the cytosol and/or a local cytosolic pH change affects RamA activity.

### The PhoQ sensor kinase protein binds cationic antimicrobial peptides to regulate virulence gene expression

The PhoPQ two-component system is a key regulator of virulence in enteric pathogens, consisting of the inner membrane sensor PhoQ and the cytosolic response regulator PhoP [98]. The sensor PhoQ detects multiple signals, including low periplasmic magnesium, periplasmic unsaturated long-chain fatty acids and cationic antimicrobial peptides (cAMPs), and cytosolic acidic pH [87,99–101]. Upon sensing one or more of these signals, PhoQ becomes autophosphorylated and transfers the phosphate group to its cognate partner PhoP. The phosphorylated response regulator PhoP then activates the transcription of genes involved in virulence and survival within the host [98].

**Fig 3:**
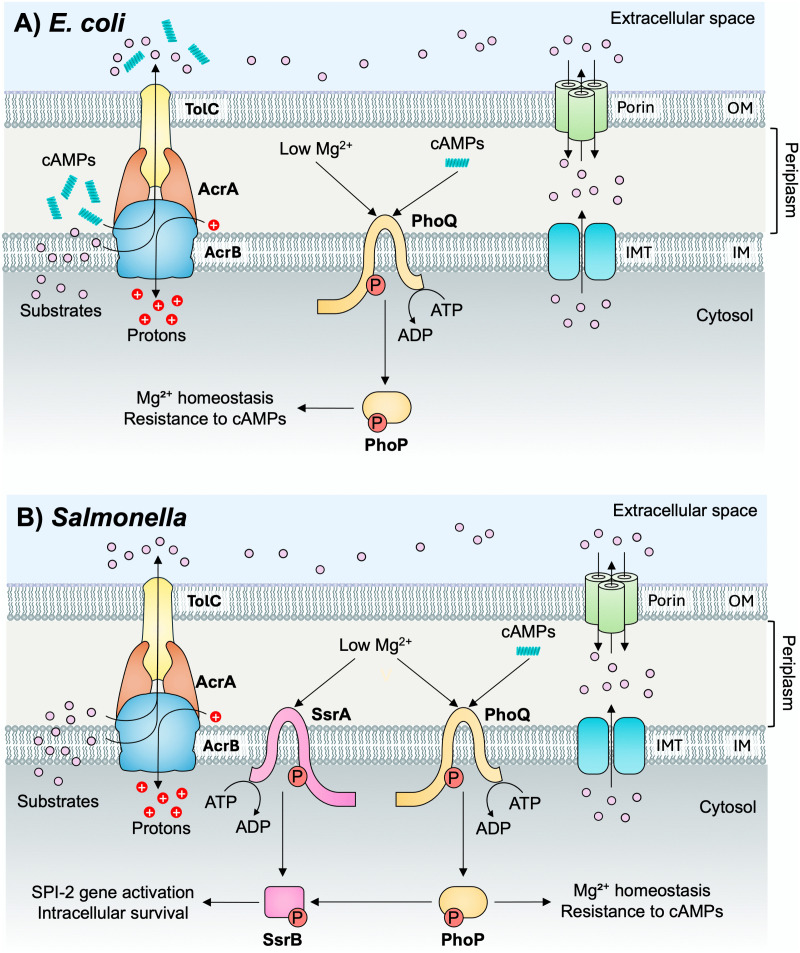
Schematic diagram of the potential mechanism of differential AcrAB-TolC modulation of PhoPQ in *Escherichia coli* versus *Salmonella.* **A)** In *E. coli* and *Salmonella*, PhoQ responds environmental signals, such as low Mg²^+^, acidic pH, and cAMPs, to phosphorylate PhoP, driving genes involved in Mg²^+^ homeostasis and AMP resistance. In *E. coli*, AcrAB-TolC exports cAMPs and substrates while facilitating proton flux, limiting prolonged PhoQ activation, and supporting adaptation rather than virulence as it lacks SPI-2. Dashed lines indicate a possible but unproven link. **B)** In *Salmonella*, the PhoQ sensor kinase is activated by host cell signals including periplasmic low Mg²^+^ and cAMPs and acidic cytosolic pH. Upon activation, PhoQ autophosphorylates and transfers the phosphate to PhoP, which induces genes required for intracellular survival, including *ssrAB*, encoding the *Salmonella* Pathogenicity Island-2 (SPI-2) regulator SsrAB. SsrA responds to low Mg²^+^ and acidic pH to phosphorylate SsrB, which can also be activated directly by acidic pH. Phosphorylated SsrB induces SPI-2 genes, promoting intracellular survival and systemic infection. Proton translocation by AcrAB-TolC possibly supports SsrB activation, while the failure to export cAMPs likely allows sustained PhoQ sensing of the intracellular environment, ensuring appropriate virulence gene expression. IMT, inner membrane transporter; IM, inner membrane; OM, outer membrane.

In *E. coli*, AcrAB-TolC has been demonstrated to export cAMPs [32]. However, in *S.* Typhimurium*,* attempts to show that AcrAB-TolC exports cAMPs have not been successful; instead, ATP-binding cassette transporters have been implicated in cAMP export [102,103]. Whether this holds true for *E. coli* is not known since the export of cAMPs by other tripartite ATP-dependent systems has not apparently been tested. These observations are intriguing because *Salmonella* use cAMPs as a signal that they are within a host and need to regulate virulence genes accordingly [87,104]. Damage to the outer membrane by cAMPs permits their access to the periplasm, where they bind to the PhoQ sensor kinase to initiate a program of virulence gene transcription via the PhoP response regulator [105]. Thus, excess efflux of cAMPs could, in this case, interfere with the ability of a pathogen to detect the host microenvironment and stimulate virulence (Fig 3).

Conversely, upon loss of RND efflux function, certain molecules can accumulate within the periplasm and be sensed by periplasmic sensor proteins, triggering the dysregulation of virulence gene expression. In *V. cholerae*, the deletion of all six RND efflux systems has been suggested to cause a periplasmic buildup of metabolites, thereby activating ToxR and other two-component systems to trigger adaptive responses and modulate virulence [106]. However, the simultaneous deletion of all RND efflux systems likely causes substantial physiological stress, complicating the interpretation of whether the observed regulatory effects are direct consequences of altered substrate accumulation or indirect effects of global envelope perturbation. Altered RND efflux activity may also intersect with envelope stress pathways, such as CpxAR, Rcs, or σ^E^ systems, which are known to influence virulence regulatory networks [107]. Thus, the integration of RND efflux activity with sensory regulatory systems suggests that RND efflux pumps may have evolved not only as detoxification systems, but also as modulators of environmental signal perception.

## Hypothesis 3—Export of bacterial-derived substrates that alter the host microenvironment

In pathogenic bacteria, exported metabolites modulate the host immune response to facilitate pathogen survival [108]. There is evidence that RND efflux pumps contribute to the export of certain bacterial factors that manipulate the host microenvironment, including siderophores, quorum-sensing molecules, and toxins, to manipulate the host and ultimately establish an infection.

### Siderophores

The host environment has scarce soluble iron, an essential nutrient for bacterial metabolism and growth [109]. Therefore, bacteria secrete siderophores, high-affinity iron-chelating molecules, to scavenge iron from their environment and produce soluble iron complexes [110]. In addition to capturing iron, some siderophores regulate the production of virulence factors [111] and influence host cells by dampening antimicrobial responses towards invading pathogens [112,113]. While some siderophores have specific exporters, there is evidence to suggest that RND efflux pumps are also involved in the secretion of siderophores. Emergent themes include that siderophores can be secreted from multiple RND efflux pumps by a single organism, and that two-component regulatory systems are involved in the detection of failed siderophore export and subsequent RND efflux pump induction.

In *E. coli,* multiple RND efflux pumps play roles in the export of siderophores such as enterobactin, which is found in *E. coli*, *S. enterica*, and several other Enterobacterales [114–116]. Horiyama and Nishino [117] showed that deletion of three RND efflux loci (*acrAB*, *acrD*, and *mdtABC*) significantly reduced enterobactin secretion, which was rescued by complementation with any of the individual genes [117]. In contrast, *E. coli* lacking only one or two RND efflux genes had slight or no reductions in enterobactin secretion, suggesting functional overlap in siderophore export across RND efflux pumps. Similarly, in *S. maltophilia*, a Pseudomonadota opportunistic pathogen, secretion of the siderophore stenobactin is mediated by both SmeYZ and SmeDEF RND efflux pumps. Wu and colleagues [118] found that deletion of individual pumps only slightly reduced stenobactin secretion, whereas the deletion of both RND efflux pumps significantly impaired stenobactin secretion. These data suggest that in diverse pathogens, multiple RND efflux pumps can secrete siderophores.

The involvement of RND efflux pumps in siderophore export can be linked to the activation of pump expression by two-component regulatory systems that sense and respond to changes in the microenvironment. A well-fleshed-out example is the *V. cholerae* VexGH RND efflux pump and the siderophore vibriobactin, which contribute to the acquisition of environmental iron [119]. The Bina laboratory demonstrated that inactivation of *vexGH* RND impaired vibriobactin secretion, reduced growth in iron-limited media, and activated the CpxRA two-component stress response system [120,121]. Their findings suggest that VexGH secretes vibriobactin and that, in turn, the Cpx system activates *vexGH* expression [120,121]. This reciprocal relationship between RND efflux pumps and two-component regulatory systems [106] reveals that deep connections between substrate export and environmental sensing contribute to bacterial maintenance of cellular homeostasis.

### Quorum-sensing molecules

Quorum sensing (QS) is a cell-to-cell communication system that allows bacteria to modulate gene expression in response to fluctuations in cell density [122]. Bacteria that use QS produce and secrete signaling molecules, known as autoinducers, which increase in concentration with cell density. When the autoinducer concentration reaches a critical threshold, it triggers changes in gene expression associated with antibiotic resistance, biofilm formation, and virulence [123]. In addition to modulating bacterial behaviors, QS molecules can also impact host cells. For example, *N-*acyl-homoserine lactones (AHLs), the main class of autoinducers in Gram-negative bacteria, stimulate apoptosis in various types of mammalian cells and modulate immune signaling pathways in macrophages [124,125]. As such, QS molecules are considered virulence factors that modulate bacteria and often the host microenvironment.

While *Pseudomonas aeruginosa* encodes multiple RND efflux pumps, there is accumulating indirect evidence that long-chain AHLs, such as *N*-(3-oxododecanoyl)-L-homoserine lactone (3OC_12_-HSL), are actively exported by MexAB-OprM. Evans and colleagues [126] reported that overexpression of *mexAB-oprM* due to a *nalB* mutation showed reduced 3OC_12_-HSL-dependent activation of QS-regulated virulence factors, which was restored to wild-type levels upon deletion of the *mexAB-oprM* operon. Similarly, Minagawa and colleagues [127] found that *mexB* deletion enhanced QS responses, including increased *lasB* promoter-driven GFP expression and LasB elastase activity, while complementation with wild-type *mexB*, but not the substrate-binding pocket mutant *mexB (*D681A*)*, suppressed these responses. Although based on reporter assays, these results imply a role for MexAB-OprM in 3OC_12_-HSL efflux. More direct evidence was provided by Pearson and colleagues [128], who showed that dissipation of the proton motive force with sodium azide led to increased intracellular accumulation of tritium-labeled 3OC_12_-HSL, but not *N*-butyryl-HSL (C_4_-HSL). Additionally, 3OC_12_-HSL accumulated to higher levels in the *mexAB-oprM* mutant and was reduced to wild-type levels upon complementation, confirming that a functional MexAB-OprM efflux pump is required for its export. In contrast, C_4_-HSL accumulation was unaffected, suggesting that short-chain AHLs exit the cell via passive diffusion, while long-chain AHLs require active efflux through RND transporters, particularly MexAB-OprM.

As in *P. aeruginosa*, *Burkolderia* species also appear to use RND efflux pumps to export QS molecules. For instance, in *B. cenocepacia*, Buroni and colleagues found that supernatants from strains lacking RND transporter genes *amrB* or *bpeB* induced ~30% less of an *E. coli* reporter gene responsive to *N*-octanoyl homoserine lactone (C_8_-HSL) compared to the wild-type strain, suggesting reduced AHL export [129]. However, there is no direct quantification of AHL secretion by RND efflux pumps in *B. cenocepacia*. In contrast, in *B. pseudomallei*, Chan and colleagues [130] found that a mutant strain lacking *bpeAB* (encoding RND inner membrane transporter BpeB and periplasmic adaptor protein BpeA) was defective in extracellular secretion of AHLs. The *bpeAB* mutant strain also showed significantly higher intracellular accumulation of exogenously added carbon-14 labeled AHLs compared to wild-type [130]. To ascertain which AHLs were exported by BpeAB, reversed-phase high-performance liquid chromatography was used to profile six long-chain AHLs from wild-type and *bpeAB* mutant supernatants. Unlike the wild-type strain, the *bpeAB* mutant secreted none of the six detectable AHLs into the culture medium [130]. This data suggests that the BpeAB-OprB is likely involved in export of AHLs in *B. pseudomallei*.

### Exotoxins

Exotoxins are used by bacterial pathogens to damage host cells and evade the host immune system. They are soluble proteins of varying sizes that are secreted into the extracellular environment. There is no direct evidence for toxin export by the transporter components of RND efflux pumps, but the outer membrane channel TolC appears to be capable of exporting toxins in complex with other transporter families. For example, MacAB-TolC is a tripartite ATP family efflux pump that exports heat-stable enterotoxin II in *E. coli* [131]*.* Other tripartite type 1 secretion systems, such as HlyBD-TolC and CvaAB-TolC, also export proteinaceous toxins using ATP [132]. Since RND efflux pumps can export peptides (e.g., cAMPs), they may be able to export peptide toxins, but likely not large proteins or folded proteins.

## Hypothesis 4—RND efflux pump activity and membrane energetics

RND efflux pumps utilize energy derived from the translocation of protons from the periplasm to the cytosol to export substrates. Therefore, proton movement and substrate export are inseparable in RND efflux pumps. The import of protons inherently changes energetics across the inner membrane [133], and could do so in a manner that supports virulence. Conversely, there appear to be environments during infection where RND efflux pumps are selected against, potentially because their effects on membrane energetics interfere with virulence. In this section, we speculate that RND efflux pump activity could affect membrane energetics in ways that bear on infection.

### Do RND efflux pumps reduce ATP accumulation during infection?

It is feasible that during infection, there are specific microenvironments in which the movement of protons into the cytosol by RND efflux pumps is needed to counterbalance other cellular processes that export protons. For example, proton import could balance the electron transport system under conditions in which bacteria use oxidative phosphorylation. *Salmonella* uses oxidative phosphorylation early in the gut to outcompete other microbes. As the gut becomes inflamed in response to *Salmonella* infection, there is an increase in the availability of oxygen, which is then consumed by *Salmonella* [134,135]. Furthermore, the expression of *acrA*, *acrB*, and *tolC* in *S.* Typhimurium is increased in response to oxygen shock [136]. Therefore, we speculate that RND efflux pumps, which are required for colonization of the gut, reduce proton motive force, and thereby contribute to limiting ATP accumulation. Another microenvironment in which reduction of ATP production could be important is the macrophage phagosome, a low pH and low magnesium vesicle in which high cytosolic ATP becomes toxic in part due to sequestration of available magnesium. To counteract this, *Salmonella* produces the MgtC protein, which inhibits the ATP synthase, enabling proton accumulation in the periplasm [137]. Under these circumstances, the RND import of protons into the cytosol could reduce the availability of periplasmic protons for ATP production and/or contribute to the maintenance of cytosolic pH balance. Accordingly, the expression of *acrA*, *acrB*, and *tolC* in *S.* Typhimurium increases during infection of murine macrophages [136]. The *S. enterica* MacAB-TolC ATP-dependent efflux pump active within phagosomes could also contribute to a reduction in cytosolic ATP in a manner that supports virulence [138]. A potential role for tripartite efflux pumps in limiting the accumulation of ATP during infection remains to be directly measured.

### Do RND efflux pumps transfer protons to cytosolic signaling molecules?

**Fig 4:**
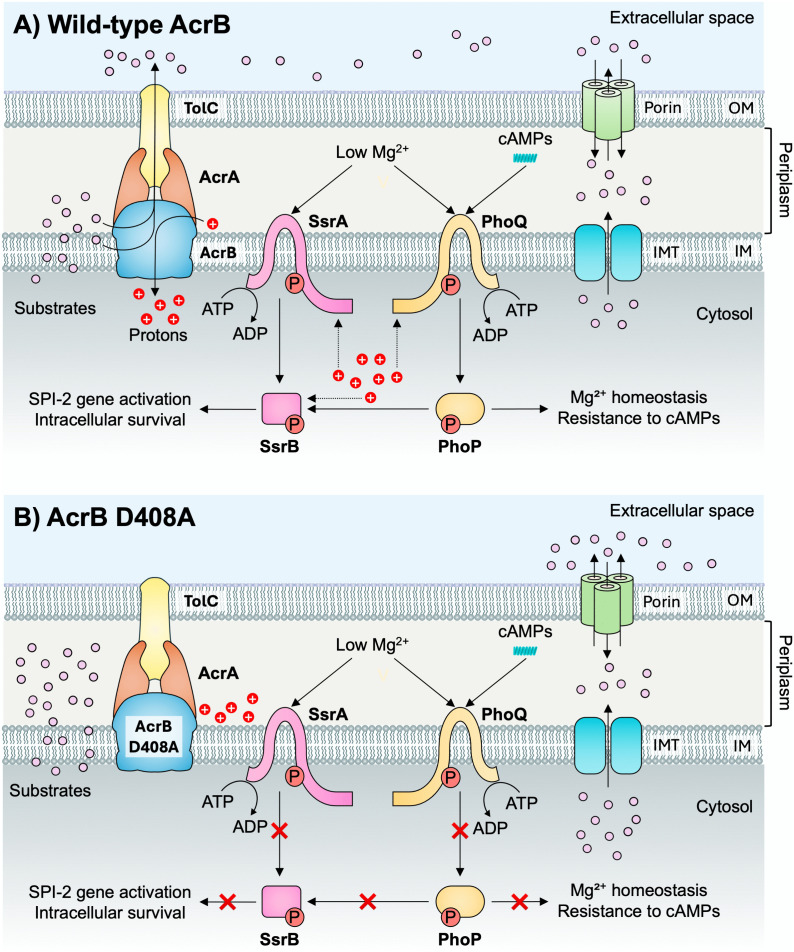
Schematic diagram of how proton translocation through AcrB may enhance *Salmonella* Pathogenicity Island 2 (SPI-2) activation in *Salmonella.* **A)** During infection of host cells or in SPI-2 inducing media consisting of signals, such as low Mg²^+^, periplasmic acidic pH, and cAMPs, the sensor kinases PhoQ and SsrA are activated and autophosphorylate, leading to the phosphorylation of response regulators PhoP and SsrB, respectively. PhoP induces the expression of *ssrAB*, while phosphorylated SsrB drives SPI-2 gene expression required for intracellular survival. Proton translocation through the AcrAB-TolC efflux pump likely contributes to optimal activation of the SsrAB system, thus promoting SPI-2 expression. **B)** In the AcrB D408A mutant, proton translocation through AcrAB-TolC is impaired, therefore, efflux cannot occur and substrates accumulate within the cell. The loss of proton translocation likely diminishes PhoP and SsrAB activation, leading to decreased SPI-2 gene expression and impaired intracellular survival. Dashed lines indicate a possible but unproven link. IMT, inner membrane transporter; IM, inner membrane; OM, outer membrane.

One possibility is that the protons transferred by RND efflux pumps enable localized protonation of virulence determinants. For instance, the *S. enterica* cytosolic domain of the PhoQ sensor kinase is not only phosphorylated but also protonated upon activation of the macrophage phagosome, which undergoes a pH drop from neutral to approximately 5.5 after phagocytosis [101,139]. Similarly, the response regulator of the SsrAB two-component system, SsrB, is protonated upon activation in the phagosome, which changes its conformation and enables virulence gene expression [140,141]. While it has been suggested that AcrAB-TolC mediates localized changes in membrane proton motive force [142], whether direct transfer of protons from RND efflux pumps to signaling proteins, such as PhoQ and SsrB, occurs is not known. For example, it could be interesting to determine the protonation status of PhoQ and SsrB in the D408A AcrB mutant, which assembles into a stable efflux pump protein but is unable to transfer protons to the cytosol [80,133]. Thus, it is feasible that in some circumstances export per se is not the only or the critical function of an RND efflux pump, and that protonation of signaling proteins is their key role (Fig 4).

### Could RND efflux pumps reduce fitness in specific conditions?

The notion that RND efflux pump proton transfer affects fitness is suggested by observations that in some pathogens, they are selected against under acidic conditions, suggesting that importing protons and lowering cytosolic pH may not be beneficial. In *E. coli*, growth under conditions with partial depletion of the proton motive force has been reported to select against strains with AcrAB-TolC. At pH 5.5 combined with the proton motive force uncoupler CCCP, or at pH 8, strains with AcrAB‑TolC have reduced fitness compared to strains lacking efflux pump subunits [143]. From pH 5.5–8.0, *acrA* transcript levels increased during log phase, but efflux pump fitness effects did not correlate with expression levels. These observations suggest that the fitness cost is derived from the energy burden of RND efflux pump activity rather than from gene regulation.

*Neisseria* provides an example of the fitness cost of RND efflux pumps during infection, as its only RND efflux pump appears to be selected against in the acidic female reproductive tract. Ma and colleagues [144] used genome-wide association of clinical *N. gonorrhoeae* isolates to reveal that loss-of-function mutations in *mtrC*, encoding the periplasmic adaptor protein of MtrCDE, were observed in 12.5% of cervical isolates. Furthermore, in the closely related *N. meningitis*, loss-of-function mutations in *mtrC* were suggested to be driven by urogenital adaptation. These observations suggest that the loss of MtrCDE could, in some instances, be adaptive for cervical colonization in pathogenic *Neisseria* species, and that the associated increase in antibiotic susceptibility may not have exerted substantial selective pressure prior to the introduction of antibiotic treatment [144]. We speculate that the acidic pH of the female reproductive tract selects against RND efflux pumps due to the transport of protons into the cytosol as the RND efflux pump exports molecules. While alternative explanations are that the RND efflux pump is selected against because it is costly to produce and/or export a substrate needed by the bacterial cell, such a substrate has not been identified. In other words, maintaining cytosolic pH homeostasis could be particularly challenging for bacterial cells under these conditions, and the inactivation of RND efflux pumps likely facilitates the reduction of proton influx. However, long-term evolution experiments of *Neisseria* in acidic conditions are needed to validate this hypothesis.

## Conclusions and future directions

The contributions of RND efflux pumps to virulence across Gram-negative bacteria are multifactorial yet characterized by several common themes. The earliest recognized role of RND efflux pumps, the expulsion of molecules that compromise bacterial growth, is clearly central to supporting virulence. In addition, bacteria use RND efflux systems to export small molecules that shape their microenvironments within the host, thereby increasing permissiveness for infection. RND efflux pumps also influence the composition of the periplasm, which contains proteins that sense environmental stimuli and regulate gene expression programs associated with virulence. The coupling of substrate export to proton import further suggests potential roles for RND efflux pumps in modulating virulence through localized reductions in proton motive force or alterations in proton delivery to the cytosol; however, these possibilities remain speculative. Future efforts to understand RND efflux pump contributions to virulence will likely focus on three major themes outlined below.

One of the major gaps in knowledge concerns the molecules exported by RND efflux pumps during infection. At present, there are no techniques capable of directly tracking the export of endogenous host- or bacterial-derived substrates by RND efflux pumps during infection of host cells or whole animals. Addressing this challenge may require the development of new technologies, as distinguishing bacterial metabolites from host metabolites is inherently difficult given that many metabolites are shared between the two. One potential approach is pathogen-specific isotope labeling, which has been used to track metabolic activity and fluxes of intracellular *Salmonella* during proliferation within macrophages and epithelial cells [145]. A limitation of this strategy, however, is the rate at which mammalian cells incorporate ^13^C into host-derived molecules, which may complicate interpretation.

A second area requiring further elucidation is the signaling pathways linking substrate export by RND efflux pumps to subsequent changes in gene expression. The transcriptional effects of RND efflux pumps are likely indirect. Although RND transporters such as AcrB contain domains that extend into the cytosol, they are not expected to bind DNA directly. Instead, exported substrates may be detected by periplasmic sensor proteins, such as the sensor kinases of two-component regulatory systems. Likewise, substrates originating from or diffusing into the cytosol may be sensed by transcription factors, thereby influencing gene expression.

Finally, relatively little attention has been given to the physiological consequences of proton translocation from the periplasm to the cytosol associated with RND efflux activity. Crucial questions include: What are the local or cellular effects of RND efflux pump changes in membrane proton motive force (voltage or the proton gradient)? [133]. Do changes in cytosolic pH or the ability to generate ATP impact virulence, and if so, in which host microenvironments? Addressing these questions will likely involve introducing new tools to the field.

**Key learning points**

- RND efflux pumps help Gram-negative pathogens to survive in host environments even in the absence of antibiotics and thus contribute to virulence beyond antibiotic resistance.
- They protect bacteria from host- and/or microbiota-derived growth inhibitors such as antimicrobial peptides, bile acids/salts, and steroid hormones. It is suspected that many important substrates have yet to be identified.
- They prevent accumulation of substrates that could perturb periplasmic or cytosolic sensing pathways and thereby the regulation of virulence genes, but these pathways remain partially defined.
- They export bacterial factors that modulate the host microenvironment, such as siderophores, quorum-sensing molecules and metabolites enabling nutrient acquisition, communication or immune modulation. It is likely that additional unknown substrates support virulence.
- They may influence bacterial physiology through proton movement, potentially affecting cytosolic pH, ATP levels, membrane energetics, and infection-related signaling pathways. This is an area that remains to be explored.

**Key papers**

- Detweiler CS, Alav I. Beyond antibiotic resistance: evidence for resistance-nodulation-division (RND) efflux pumps as virulence determinants. Microbiol Mol Biol Rev. 0: e00278-24. https://doi.org/10.1128/mmbr.00278-24
- Wang-Kan X, Blair JMA, Chirullo B, Betts J, La Ragione RM, Ivens A, et al. Lack of AcrB Efflux Function Confers Loss of Virulence on *Salmonella enterica* Serovar Typhimurium. mBio. 2017;8. https://doi.org/10.1128/mBio.00968-17
- Pearson JP, Van Delden C, Iglewski BH. Active Efflux and Diffusion Are Involved in Transport of *Pseudomonas aeruginosa* Cell-to-Cell Signals. J Bacteriol. 1999;181:1203–1210.
- Whittle EE, Orababa O, Osgerby A, Siasat P, Element SJ, Blair JMA, et al. Efflux pumps mediate changes to fundamental bacterial physiology via membrane potential. mBio. 2024 [cited 24 Apr 2025]. https://doi.org/10.1128/mbio.02370-24
- Weng Y, Bina XR, Van Allen ME, Bina JE. RND-mediated efflux couples antimicrobial resistance and hypervirulence in contemporary *Vibrio cholerae*. PLoS Pathog. 2026;22: e1014031. https://doi.org/10.1371/journal.ppat.1014031
